# Potentiation of LPS-Induced Apoptotic Cell Death in Human Hepatoma HepG2 Cells by Aspirin via ROS and Mitochondrial Dysfunction: Protection by N-Acetyl Cysteine

**DOI:** 10.1371/journal.pone.0159750

**Published:** 2016-07-21

**Authors:** Haider Raza, Annie John, Jasmin Shafarin

**Affiliations:** Department of Biochemistry, College of Medicine and Health Sciences (CMHS), UAE University, Al Ain, United Arab Emirates; University of South Alabama Mitchell Cancer Institute, UNITED STATES

## Abstract

Cytotoxicity and inflammation-associated toxic responses have been observed to be induced by bacterial lipopolysaccharides (LPS) in vitro and in vivo respectively. Use of nonsteroidal anti-inflammatory drugs (NSAIDs), such as aspirin, has been reported to be beneficial in inflammation-associated diseases like cancer, diabetes and cardiovascular disorders. Their precise molecular mechanisms, however, are not clearly understood. Our previous studies on aspirin treated HepG2 cells strongly suggest cell cycle arrest and induction of apoptosis associated with mitochondrial dysfunction. In the present study, we have further demonstrated that HepG2 cells treated with LPS alone or in combination with aspirin induces subcellular toxic responses which are accompanied by increase in reactive oxygen species (ROS) production, oxidative stress, mitochondrial respiratory dysfunction and apoptosis. The LPS/Aspirin induced toxicity was attenuated by pre-treatment of cells with N-acetyl cysteine (NAC). Alterations in oxidative stress and glutathione-dependent redox-homeostasis were more pronounced in mitochondria compared to extra- mitochondrial cellular compartments. Pre-treatment of HepG2 cells with NAC exhibited a selective protection in redox homeostasis and mitochondrial dysfunction. Our results suggest that the altered redox metabolism, oxidative stress and mitochondrial function in HepG2 cells play a critical role in LPS/aspirin-induced cytotoxicity. These results may help in better understanding the pharmacological, toxicological and therapeutic properties of NSAIDs in cancer cells exposed to bacterial endotoxins.

## Introduction

Oxidative stress and inflammation have been implicated in the pathophysiology of numerous diseases such as cancer, diabetes, obesity, cardiovascular and neurological disorders [1–4]. The bacterial endotoxins, lipopolysaccharides (LPS), induce inflammatory and oxidative/nitrosative stress associated toxic responses in vitro and in vivo [5, 6, 7]. LPS stimulates the production of cytokines and prostaglandin E2 (PGE2) leading to increased inflammatory response.

It also induces cytotoxicity through the production of reactive oxygen species (ROS) and reactive nitrogen species (RNS) [8,9]. Studies by Xu et al. [10] have suggested that the multiple pharmacological effects of acetylsalicylic acid (ASA, aspirin), a potent inhibitor of cyclooxygenase (COX) enzyme and a commonly used anti-inflammatory drug, may not be associated with its COX inhibitory activity.

Our previous studies have indicated increased oxidative stress, altered glutathione metabolism as well as mitochondrial dysfunction in aspirin-treated mouse macrophages and human hepatoma HepG2 cells [11–13]. Although aspirin has been established as an anti-inflammatory and anti-tumor drug, studies indicate multiple pathways including prostaglandin inhibition and activation of NF-κB as the pathways responsible for regulation of redox metabolism, cell signaling and mitochondrial functions [14–15]. Aspirin, has been shown to stimulate TNF-α-dependent necrotic inflammatory responses in cells under in vitro and in vivo conditions. Our recent studies on acetaminophen (APAP)-induced cytotoxicity using macrophages and HepG2 cells have demonstrated that these two cell lines exhibit differential responses towards APAP. Macrophages appear to be highly sensitive towards APAP exposure than HepG2 cells as observed by the degree of ROS production, oxidative stress-induced alterations in redox metabolism and mitochondrial functions [16–17]. This differential cytotoxicity appears to be associated with the differential mechanism of drug metabolism and detoxification in these cellular systems. These studies have suggested increased sensitization of macrophages towards bacterial endotoxins. Inhibition of GSH synthesis in HepG2 cells have also been reported to enhance the sensitivity of these cells towards NSAIDs which was attenuated after the treatment of NAC [12].Our studies on the effects of ASA/LPS on HepG2 cells and macrophages have shown that HepG2 cells were more resistant to the treatment of LPS alone or in combination with ASA compared to macrophages [18]. We have also shown the oxidative stress, apoptosis and mitochondrial dysfunction caused by different doses of aspirin alone at different time intervals in HepG2 cells [11].

In our present study, we have tried to further investigate the effects of LPS alone or in combination with ASA on HepG2 cells to elucidate the combined effects of the drug and endotoxin on oxidative stress and mitochondrial dysfunction in this cellular system. In addition, we have also studied the effects of NAC on LPS alone or in combination with ASA-treated cells. This is an extension of our previous study [13] on macrophages which showed that ASA facilitated enhanced LPS-induced toxicity by enhancing cellular oxidative stress and mitochondrial dysfunction, which was attenuated on treatment with NAC. Our present results suggest sensitization of HepG2 by ASA to LPS-induced toxicity by inducing oxidative stress, resulting in mitochondrial dysfunction and metabolic stress. NAC pre-treatment, however, protected the cells from the toxicological responses.

## Materials and Methods

### Materials

Aspirin, LPS, NAC, NADPH, reduced glutathione (GSH), 1-chloro 2,4-dinitrobenzene (CDNB), cumene hydroperoxide, glutathione reductase, 5,5’-dithio bis-2-nitrobenzoic acid (DTNB), cytochrome c, coenzyme Q2, antimycin A, dodecyl maltoside, N-nitrosodimethylamine (NDMA), erythromycin and ATP bioluminescent somatic cell assay kits were purchased from Sigma (St Louis, MO, USA). 2,7-Dichlorofluorescein diacetate (DCFDA) was purchased from Molecular Probes, Inc. (Eugene, OR, USA). Kits for mitochondrial membrane potential assays were procured from R & D Systems, MN, USA. Apoptosis detection kits for flow cytometry and IL6 and TNF-α measurement kits were purchased from BD Pharmingen (BD Biosciences, San Jose, USA). HepG2 cells were purchased from American Type Culture Collection (Manassas, VA, USA). Polyclonal antibodies against beta-actin, HO-1, IκB-α, NF-κBp65, PARP, Nrf-2 and cytochrome c were purchased from Santa Cruz Biotechnology, Inc (Santa Cruz, CA, USA). Reagents for electrophoresis and Western blot analyses were purchased from Bio-Rad Laboratories (Richmond, CA, USA).

### Cell culture and treatment

HepG2 cells were grown in poly-L-lysine coated 75 cm^2^ flasks (~2.0–2.5 x 10^6^ cells/ml) in DMEM supplemented with 1% non-essential amino acids, 2 mM glutamine and 10% heat-inactivated fetal bovine serum in the presence of 5% CO_2_-95% air at 37°C as described before [11,12]. Cells were cultured to 80% confluence and then treated with 1μg/ml LPS for 24h. In some cases, the cells were also treated with 5 mM ASA for 24h alone or in combination with LPS or in the presence or absence of NAC (10 mM) for 2h prior to LPS treatment. After the treatments, cells were harvested and sub-cellular fractions, mitochondria and post-mitochondrial supernatant (PMS) were isolated for further analyses as described before [11–12, 17].

### Measurement of reactive oxygen species (ROS)

HepG2 cells were cultured in 6-well plates (1–5 x 10^5^ cells/well). After 24h, cells were treated with LPS and/or 5 mM ASA as described above. The intracellular production of ROS in control and ASA and/or LPS treated HepG2 cells was measured by FACS analysis using the cell permeable probe, DCFDA, as described before [11–13, 17]. Microscopic measurement of the reactive oxygen species produced was also done using the Olympus fluorescence microscope using the same probe. For this, cells (1–5 x 10^5^ cells/ml) were grown on cover slips, treated with ASA and LPS alone or in combination or in the presence of NAC and then incubated with 5 μM DCFDA for 30 min at 37°C. Cells were then washed with phosphate buffered saline (PBS) and fluorescence analyzed immediately.

### Apoptosis measurement after LPS/ASA treatments

The measurement of apoptosis in HepG2 cells treated with LPS alone or in combination with ASA or in the presence of NAC was performed using annexin V assay by flow cytometry as described in the vendor’s protocol (BD Pharmingen, BD Biosciences, San Jose, USA) with slight modifications as described before [13, 17].The apoptotic cells were estimated as the percentage of cells that stained positive for Annexin V-FITC while remaining impermeable to PI (AV+/PI-). This method also identified the viable cells (AV-/PI-) and cells undergoing necrosis (AV+/PI+).

### Quantitation of cytokines after treatment with LPS/ASA

Cytokines, IL6 and TNF-∝ in LPS alone and/or ASA-treated and control HepG2 cells with/without NAC were measured using ELISA kits from BD Pharmingen (BD Biosciences, San Jose, USA) as described in the vendor’s protocol. The plates were read at 450 nm using the Gen 5 ELx 800 plate reader (Wincoski,VT,USA).

### Measurement of GSH and GSH metabolism

GSH is the most important cellular antioxidant, protecting cells from oxidative stress. The GSH concentration in HepG2 cells treated with LPS and/or ASA with or without NAC was measured by the enzymatic recycling method of Tietz [19] using DTNB, NADPH and GSH-reductase as described before [13].Glutathione S-transferase (GST) activity using CDNB [20], glutathione peroxidase (GSH-Px) activity using cumene hydroperoxide [21] and GSH-reductase using oxidized glutathione (GSSG) [22] as substrates were measured by standard protocols as described before [11–13,17].

### Measurement of CYP 450-dependent enzyme activities

Activities of drug metabolizing enzymes, CYP 2E1 and CYP 3A4 in control and LPS/ASA-treated HepG2 cells in the presence/absence of NAC were measured using standard substrates, N-nitrosodimethylamine and erythromycin, respectively according to the methods described earlier [23,24].

### Measurement of mitochondrial membrane potential (MMP)

The mitochondrial membrane potential in HepG2 cells treated with LPS/ASA was measured according to the vendor’s protocol (DePsipher^TM^, R &D Systems Inc.) by flow cytometry using a fluorescent cationic dye as described before [13]. The method is based on the principle that DePsipher, a cationic dye, has the property of aggregating upon membrane polarization forming an orange-red fluorescent (absorption/emission 585/590 nm) compound. If the membrane potential is reduced, the dye cannot access the transmembrane space and remains in its green fluorescent (510/527 nm) monomeric form.

### Measurement of activities of mitochondrial respiratory enzyme complexes

Freshly isolated mitochondria (5 μg protein) from LPS/ASA-treated HepG2 cells with or without NAC were suspended in 1.0 ml of 20 mM KPi buffer, pH 7.4, in the presence of the detergent, lauryl maltoside (0.2%). NADH ubiquinone oxidoreductase (Complex I) and cytochrome c oxidase (Complex IV) were measured using the substrates coenzyme Q2 and reduced cytochrome c, respectively by the methods of Birch-Machin and Turnbull [25] as described before [13].

### Measurement of ATP content

The ATP content in HepG2 cells treated with LPS/ ASA with or without NAC was determined using the ATP Bioluminescent cell assay kit according to the manufacturer’s protocol (Sigma, St Louis, MO) and samples were read using the TD-20/20 Luminometer (Turner Designs, Sunnyvale, CA).

### SDS-PAGE and Western blot analysis

Protein (50–100 μg) from the different sub-cellular fractions of control and treated HepG2 cells were electrophoretically separated on 12% SDS-PAGE [26] and transferred onto nitrocellulose membrane by Western blotting [27] Transferred proteins were then probed with primary antibodies against HO-1, IκB-∝, NF-κB, cytochrome c, Nrf-2 and PARP. Actin immunostaining, as a loading control, was used to confirm equal loading of the protein. Immunoreactive bands were visualized using the appropriate conjugated secondary antibodies. After development of the blots, the bands were visualized and quantitated using the Typhoon FLA 9500 system (GE Healthcare, Uppsala, Sweden) and expressed as relative intensity (R.I) compared to the untreated control.

### Statistical analysis

Values shown are expressed as mean ± SEM of 3 individual experiments. Statistical significance of the data was assessed using SPSS software (version 21) by analysis of variance followed by Dunnett’s post-hoc analysis. P values ≤ 0.05 were considered statistically significant.

## Results

### LPS/ASA-induced ROS production and attenuation by NAC

**Fig 1:**
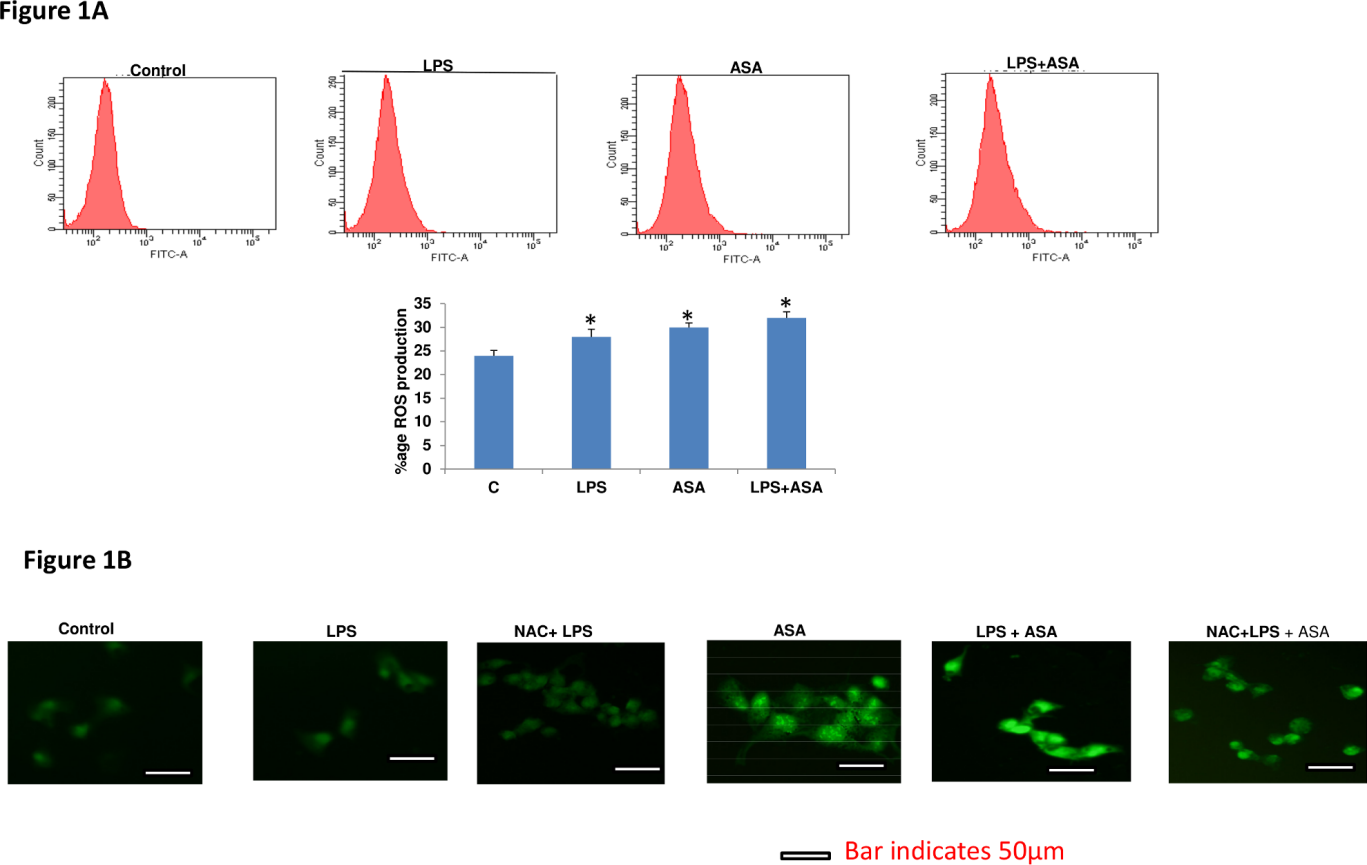
Effects of LPS and ASA on ROS production. HepG2 cells were cultured to 80% confluence and treated with 1μg/ml LPS for 24h and/or 5 mM ASA for 24h, as described in the Materials and Methods. DCFDA was used as the probe to measure ROS production in the cell lysates using DCFDA and the fluorescence generated was measured by using the FACS Canto II flow cytometer (1A) [13]. Results are expressed as a typical representation of three determinations. Bar diagram represents percentage ROS production in the control and LPS and/or ASA treated cells. Results are expressed as mean +/- SEM of at least three experiments. Asterisks indicate significant difference (* p ≤0.05) from control (C). In some cases, cells grown on cover-slips were treated with LPS and/or ASA for 24h in the presence or absence of NAC (10 mM) for 2h prior to LPS treatment and DCFDA-induced ROS fluorescence was immediately analyzed microscopically using an Olympus fluorescence microscope (1B). Typical results from control and LPS alone or in combination with ASA with or without NAC treatment from three experiments are shown.

Fig 1A shows the effects of LPS and ASA or a combination of both, on ROS production in HepG2 cells. A significant increase in ROS production was observed with LPS and ASA treatment, which further increased when a combination of the treatments was used suggesting a synergistic effect on ROS production in HepG2 cells when LPS and ASA are combined. Microscopic studies confirmed an increased ROS production in LPS and/or ASA-treated HepG2 cells (Fig 1B). A significant reduction was observed on NAC pre-treatment.

### Effect of NAC on LPS and ASA-induced apoptosis

**Fig 2:**
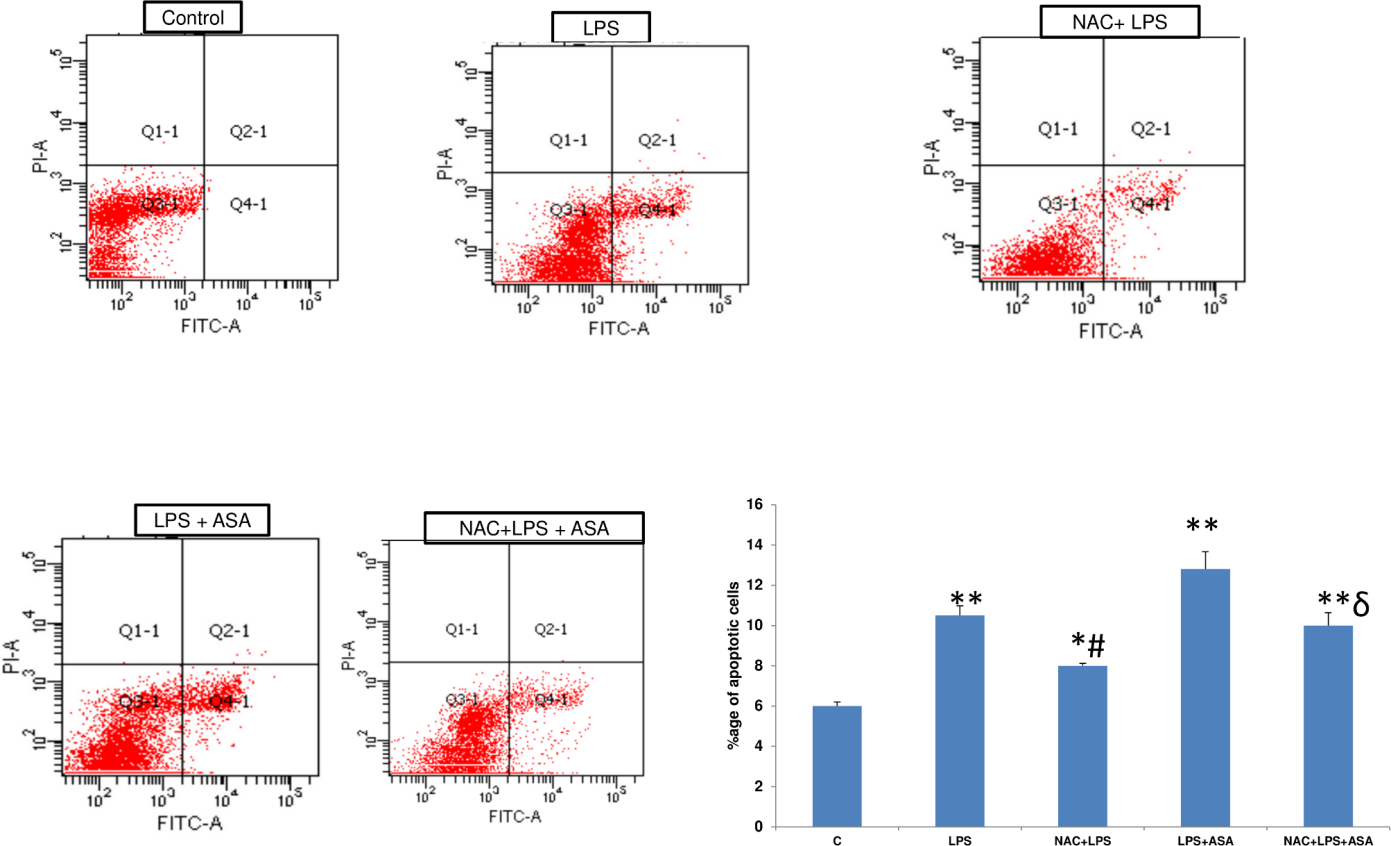
LPS- and ASA-induced apoptosis and protection by NAC. HepG2 cells were cultured to 80% confluence and treated with LPS alone (1μg/ml) for 24h or in combination with 5 mM ASA for 24h or in the presence of NAC (10 mM) for 2h prior to LPS treatment as described in the Materials and Methods. Apoptosis was measured by flow cytometry using FACSDiva software as described before [13]. Representative dot plots are shown. Histogram shows %age apoptotic death from a representative result expressed as mean +/- SEM of at least three experiments. Asterisks indicate significant difference (*p≤0.05; ** p ≤0.01 from control (C), ≠p ≤0.05 compared to LPS-treated cells and ɗ p≤0.05 compared to LPS- and ASA-treated cells).

A significant (>60%) increase in apoptotic cell death in HepG2 cells was observed after treatment with LPS alone (Fig 2) which further increased (~ 2-fold) when a combination of LPS and ASA treatment was used. ASA alone has also shown to cause increased apoptosis as observed before [11]. On the other hand, a marked reduction in ROS production was observed on NAC pre-treatment though the level was significantly higher than that in the control untreated cells. These results indicate that NAC treatment has only partially inhibited the production of ROS in HepG2 cells suggesting the selectivity of ROS production from multiple sources in these cells.

### LPS and ASA-induced cytokine production

**Fig 3:**
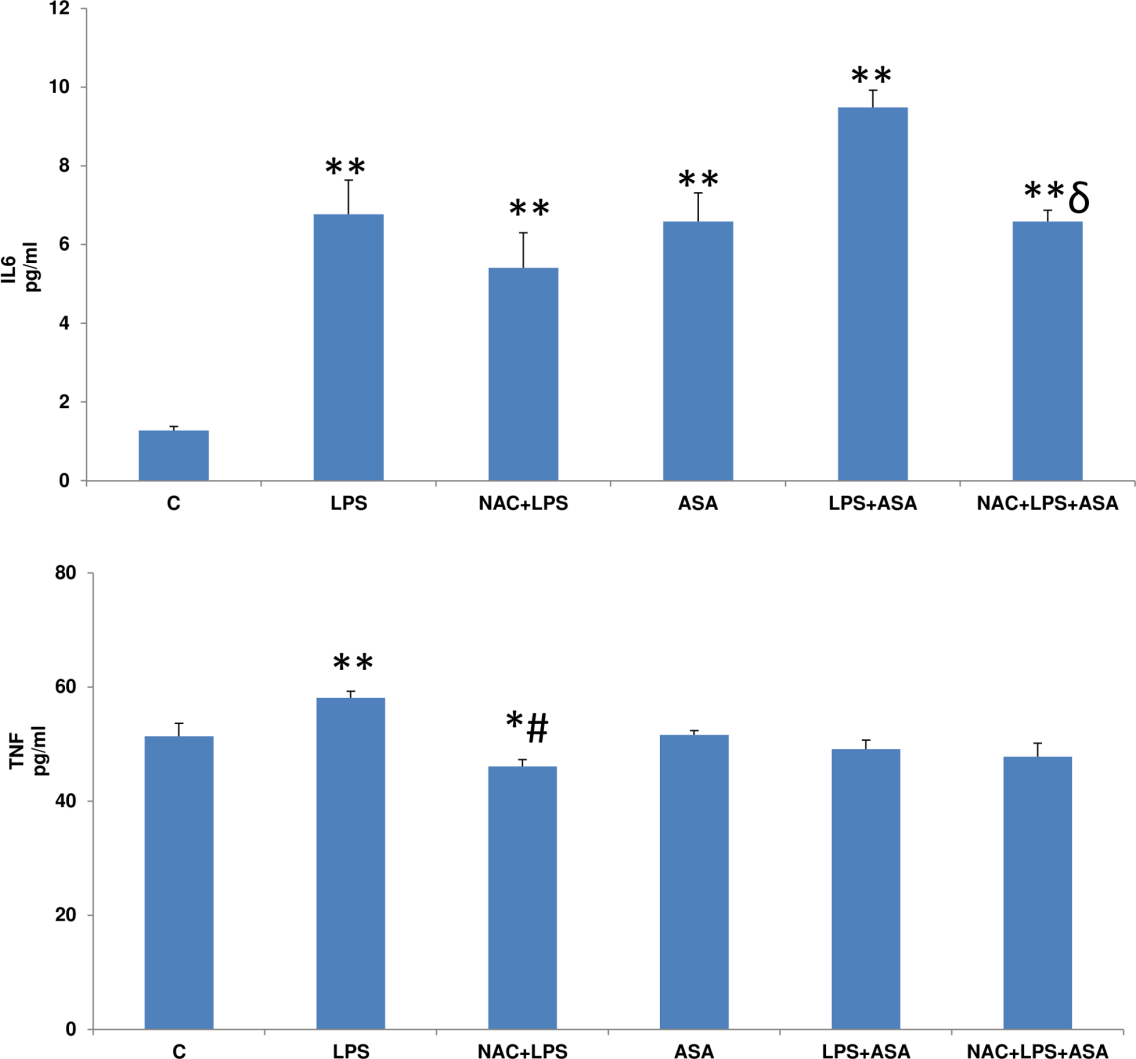
Quantitation of cytokines’ production after LPS and ASA treatments. HepG2 cells cultured to 80% confluence, were treated with LPS alone (1μg/ml) for 24h. In some cases, the cells were also treated with 5 mM ASA for 24h alone or in combination with LPS or in the presence of NAC (10 mM) for 2h prior to LPS treatment as described above. IL6 and TNF-α were measured using standard ELISA kits as described in Materials and Methods. Results are expressed as mean +/-SEM of at least three experiments. Asterisks indicate significant difference (*p≤0.05, **p ≤0.001 from control (C), ≠p <0.05 compared to LPS-treated cells and ɗ p≤0.05 compared to LPS- and ASA-treated cells).

The levels of cytokine, IL6 was significantly increased (~5-fold) with LPS or ASA treatments (Fig 3) which further increased (>6-fold) after the combined treatment of LPS and ASA. NAC pre-treatment significantly reduced the level though it remained significantly higher than that in the control cells. On the other hand, TNF-α was moderately but significantly increased after LPS treatment which was inhibited significantly in NAC treated cells. A combination of LPS and ASA or ASA alone did not result in any significant alteration in TNF-α production in HepG2 cells.

### GSH metabolism in LPS and ASA-treated HepG2 cells with/without NAC

**Fig 4:**
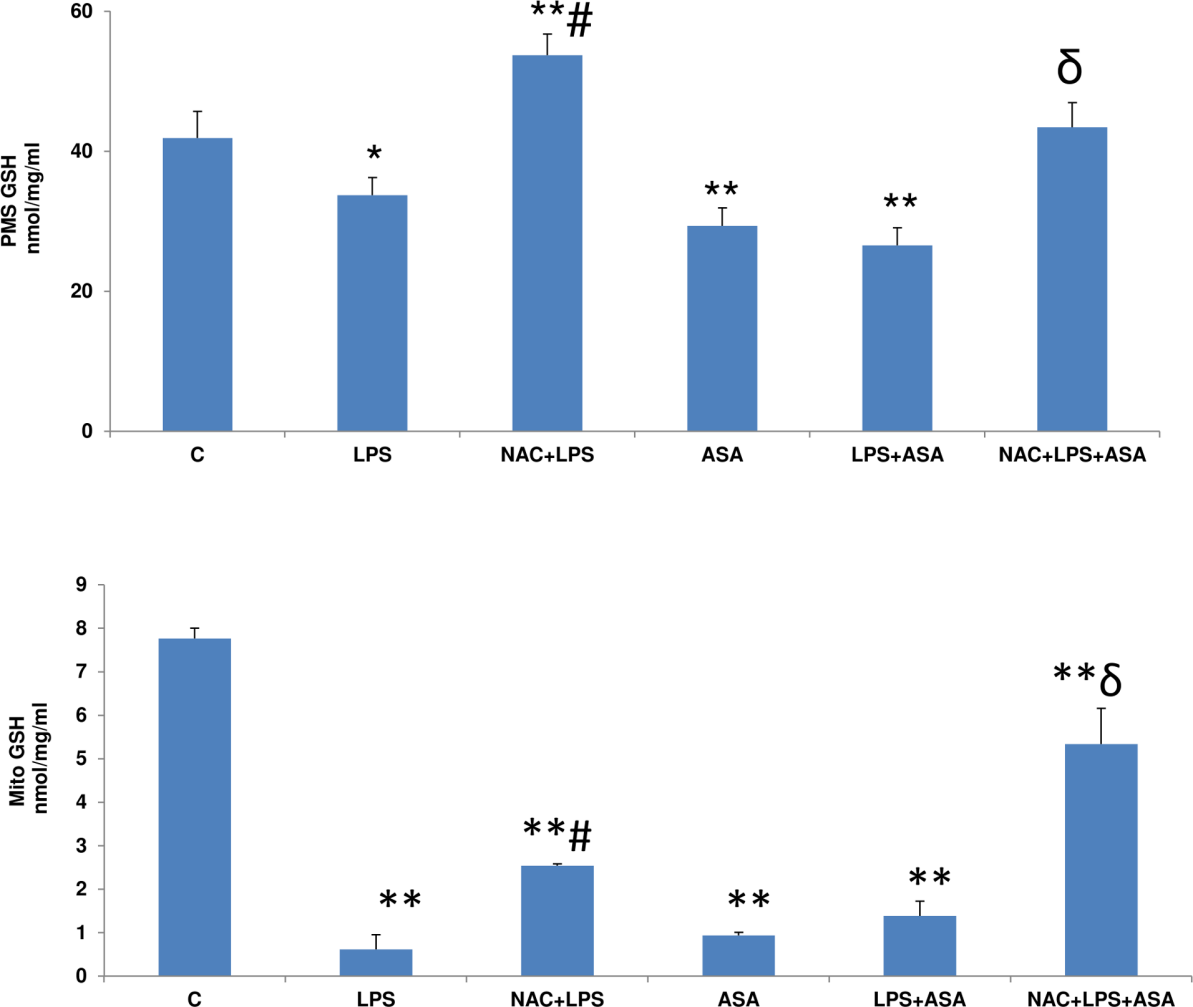
LPS- and ASA-induced alterations in GSH. GSH was measured by the enzymatic recycling method in the mitochondria and PMS isolated from cells treated with LPS alone (1μg/ml) for 24h. In some cases, the cells were also treated with 5 mM ASA for 24h alone or in combination with LPS or in the presence of NAC (10 mM) for 2h prior to LPS treatment, using glutathione reductase and NADPH as described before [13]. Results are expressed as mean +/- SEM of at least three experiments. Asterisks indicate significant difference (*p<0.05; ** p <0.01 from control (C), ≠p <0.05 compared to LPS-treated cells and ɗ p<0.05 compared to LPS- and ASA-treated cells).

A significant reduction in the total post mitochondrial GSH pool in HepG2 cells was observed after treatment with LPS or ASA alone or in combination (Fig 4). Treatment with NAC prior to the treatment with LPS and ASA markedly increased the cytosolic GSH pool. Mitochondrial GSH pool (Fig 4 lower panel), on the other hand, was reduced drastically (>80–90%) after treatment with LPS or ASA alone or in combination, which was significantly recovered after pre-treatment with NAC. However, the level of mitochondrial GSH remained significantly lower even in the NAC pre-treated cells. These results suggest differential mechanisms of protection by NAC in the cytosol and mitochondria since the mitochondrial GSH seems to be more sensitive to LPS and ASA treatments than cytosolic GSH.

**Fig 5:**
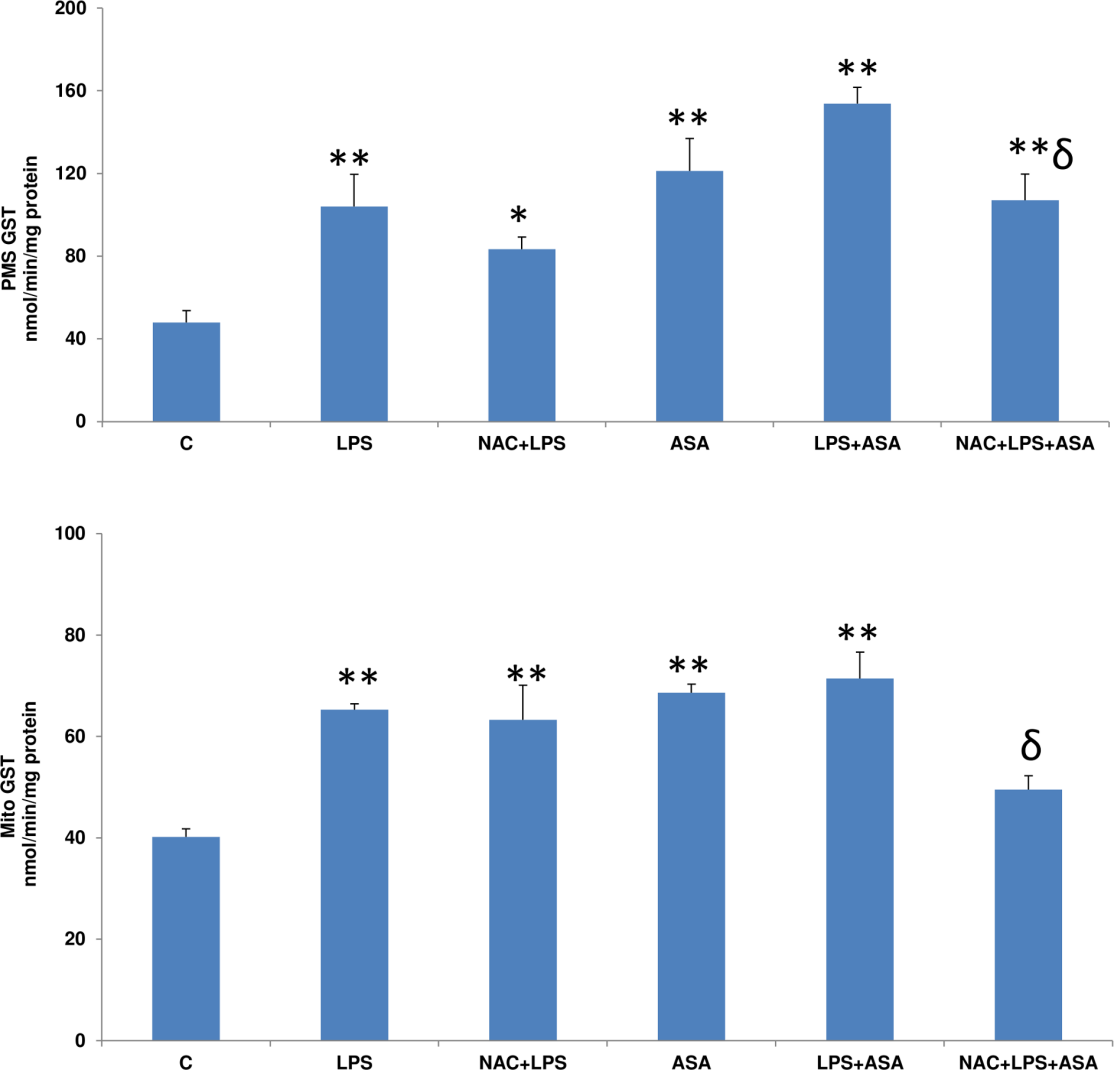
LPS- and ASA-induced effects on GST activity. HepG2 cells were cultured to 80% confluence and treated with LPS alone (1μg/ml) for 24h. In some cases, the cells were also treated with 5 mM ASA for 24h alone or in combination with LPS or in the presence of NAC (10 mM) for 2h prior to LPS treatment, as described in Materials and Methods. GST activity in the mitochondria and PMS was measured as described before [13]. Results are expressed as mean +/- SEM of at least three experiments. Asterisks indicate significant difference (*p≤0.05, **p ≤0.001 from control (C) and ɗ p≤0.05 compared to LPS- and ASA-treated cells).

The GSH conjugating activity of GST enzymes with CDNB as a substrate, both in the mitochondrial and post mitochondrial fractions, was found to be markedly increased after LPS and ASA treatments alone which further increased in combination (Fig 5). Treatment with NAC had an inhibitory effect on the activation of GSTs in these cellular compartments, which was significant when the cells were treated with LPS and ASA combined.

**Fig 6:**
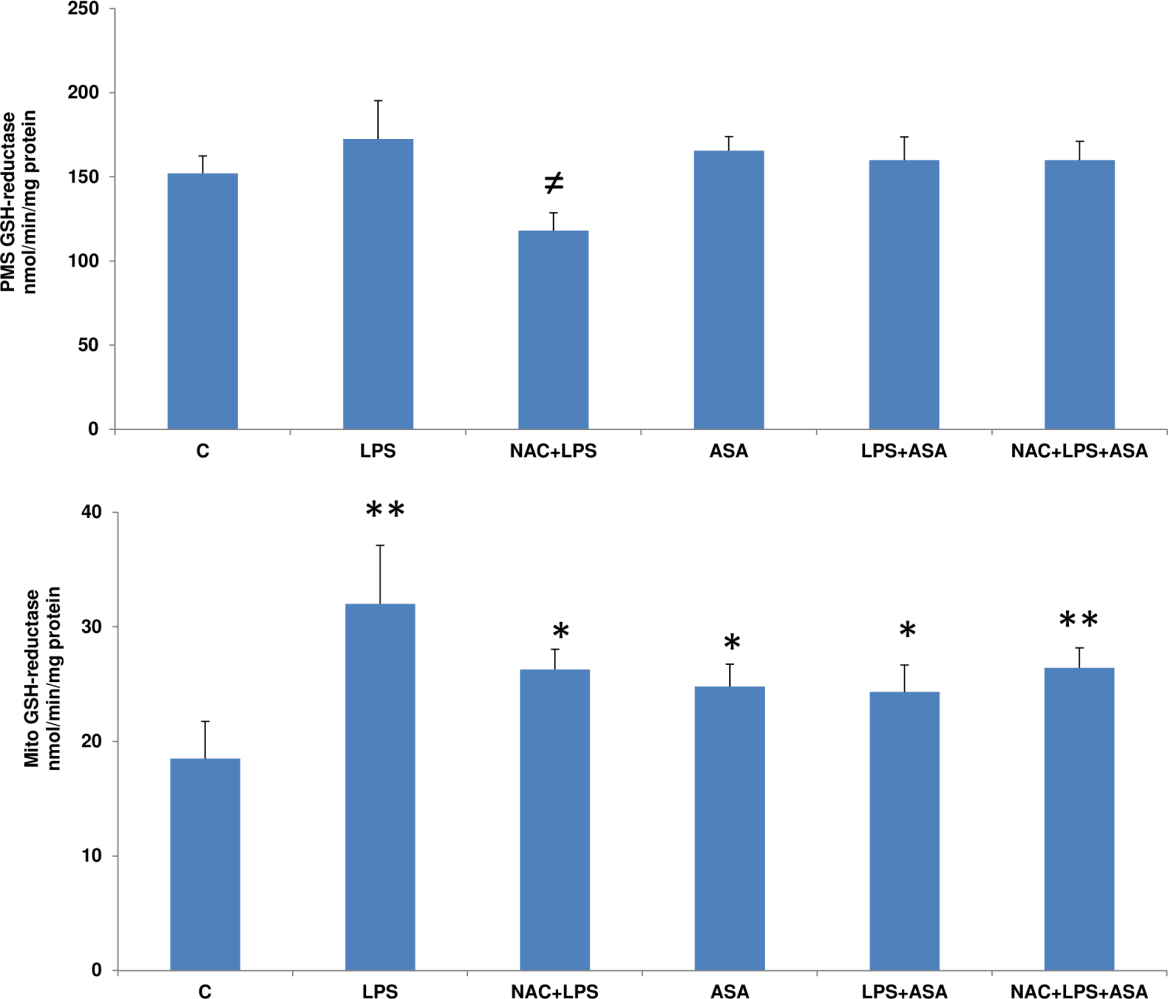
LPS- and ASA-induced alterations in GSH-reductase activity. HepG2 cells were cultured to 80% confluence and treated with LPS alone (1μg/ml) for 24h. In some cases, the cells were also treated with 5 mM ASA for 24h alone or in combination with LPS or in the presence of NAC (10 mM) for 2h prior to LPS treatment, as described in Materials and Methods. GSH-reductase activity in the mitochondria and PMS was measured using oxidized glutathione (GSSG) as substrate as described before [12]. Results are expressed as mean +/- SEM of at least three experiments. Asterisks indicate significant difference (*p≤0.05, **p ≤0.001 from control (C) and ≠p <0.05 compared to LPS-treated cells).

Post-mitochondrial GSH-reductase activity was not significantly affected after LPS or ASA treatments alone while it was significantly increased in the mitochondrial fraction (Fig 6). NAC pre-treatment, however, exhibited a significant inhibition of enzyme activity only in the cells treated with LPS alone but not in combination with ASA.

**Fig 7:**
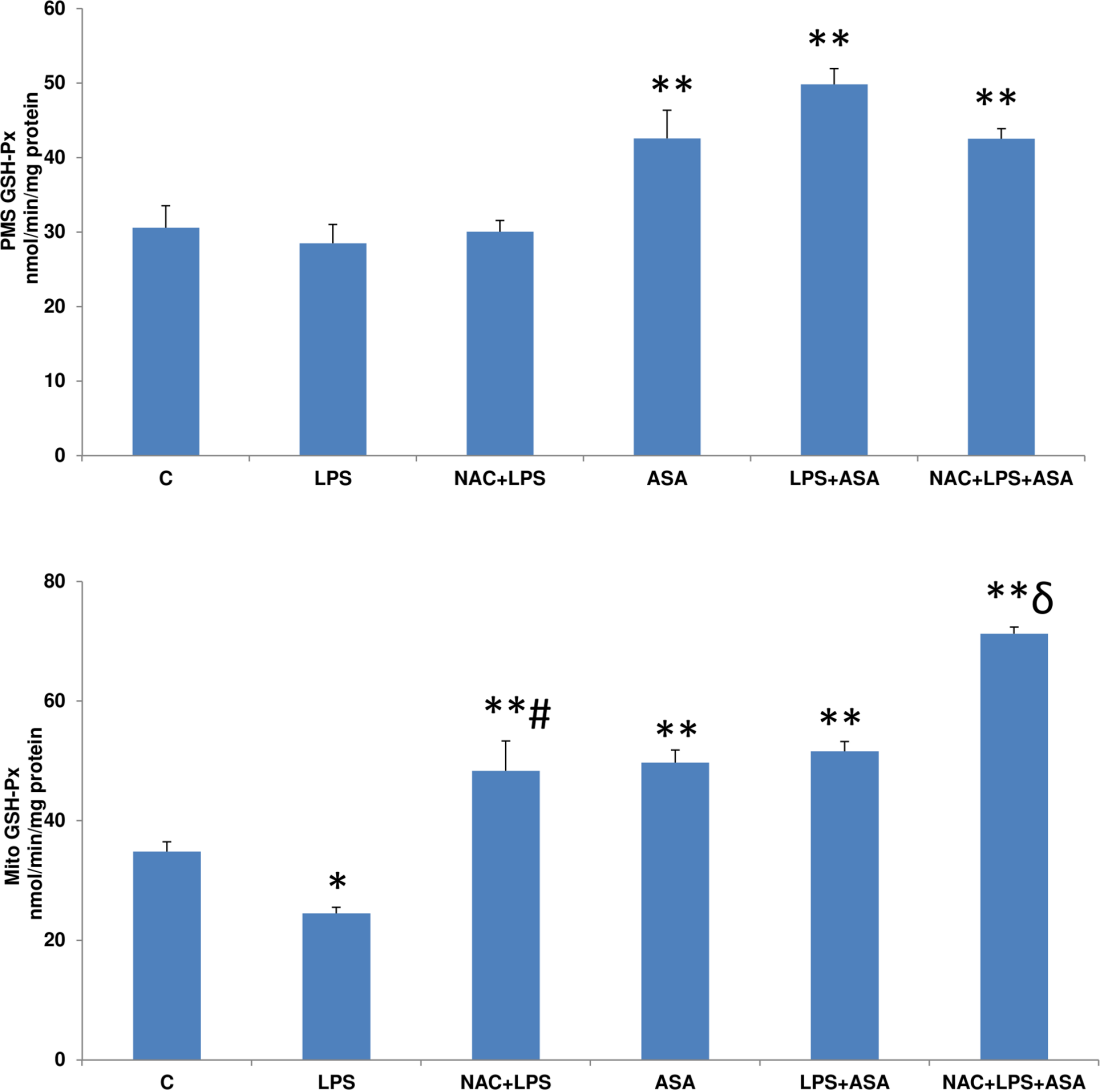
LPS- and ASA-induced alterations in GSH-Px activity. HepG2 cells were cultured to 80% confluence and treated with LPS alone (1μg/ml) for 24h. In some cases, the cells were also treated with 5 mM ASA for 24h alone or in combination with LPS or in the presence of NAC (10 mM) for 2h prior to LPS treatment, as described in Materials and Methods. GSH-Px activity in the mitochondria and PMS was measured as described before [13]. Results are expressed as mean +/- SEM of at least three experiments. Asterisks indicate significant difference (*p≤0.05, **p ≤0.001 from control (C), ≠p <0.05 compared to LPS-treated cells and ɗ p≤0.05 compared to LPS- and ASA-treated cells).

GSH-Px activity in the post-mitochondrial fraction (Fig 7) of HepG2 cells was not affected by LPS treatment alone, which however increased significantly when cells were treated with ASA alone or in combination with LPS. Pre-treatment with NAC had no appreciable effects. On the other hand, mitochondrial GSH-Px enzyme activity (Fig 7 lower panel), was inhibited by LPS treatment alone, but was significantly elevated in combination with ASA or with ASA treatment alone, which was further increased with NAC pre-treatment.

### Effects of LPS and ASA treatments on cytochrome P450 activities

**Fig 8:**
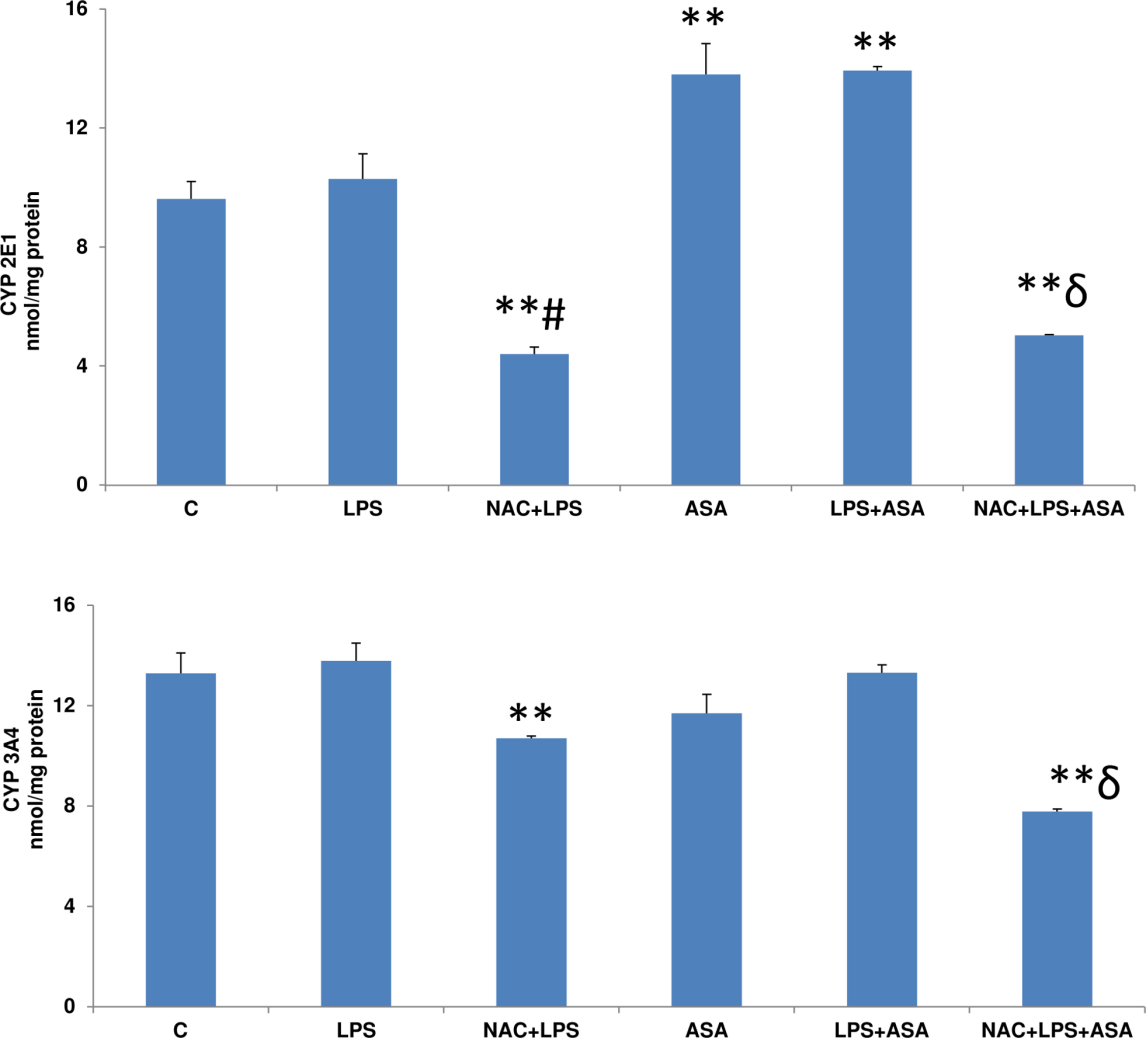
LPS- and ASA-induced alterations in CYP activities. HepG2 cells were cultured to 80% confluence and treated with LPS alone (1μg/ml) for 24h. In some cases, the cells were also treated with 5 mM ASA for 24h alone or in combination with LPS or in the presence of NAC (10 mM) for 2h prior to LPS treatment, as described in Materials and Methods. CYP2E1 and CYP 3A4 activities were measured as described in Materials and Methods. Results are expressed as mean +/- SEM of at least three experiments. Asterisks indicate significant difference (**p ≤ 0.001) from control (C), ≠p <0.05 compared to LPS-treated cells and ɗ p<0.05 compared to LPS- and ASA-treated cells).

CYP 2E1 activity increased significantly in HepG2 cells treated with LPS in combination with ASA or ASA alone compared to cells treated with LPS alone (Fig 8). NAC pre-treatment resulted in a significant inhibition of the enzyme activity suggesting NAC may prevent the CYP2E1-dependent activity implicated in ROS production and oxidative stress.

CYP 3A4 activity, on the other hand, was not affected when cells were treated with LPS or ASA alone or in combination (Fig 8 lower panel). NAC pre-treatment, however, inhibited the enzyme activity.

### Effects of NAC on mitochondrial functions in LPS and ASA-treated cells

**Fig 9:**
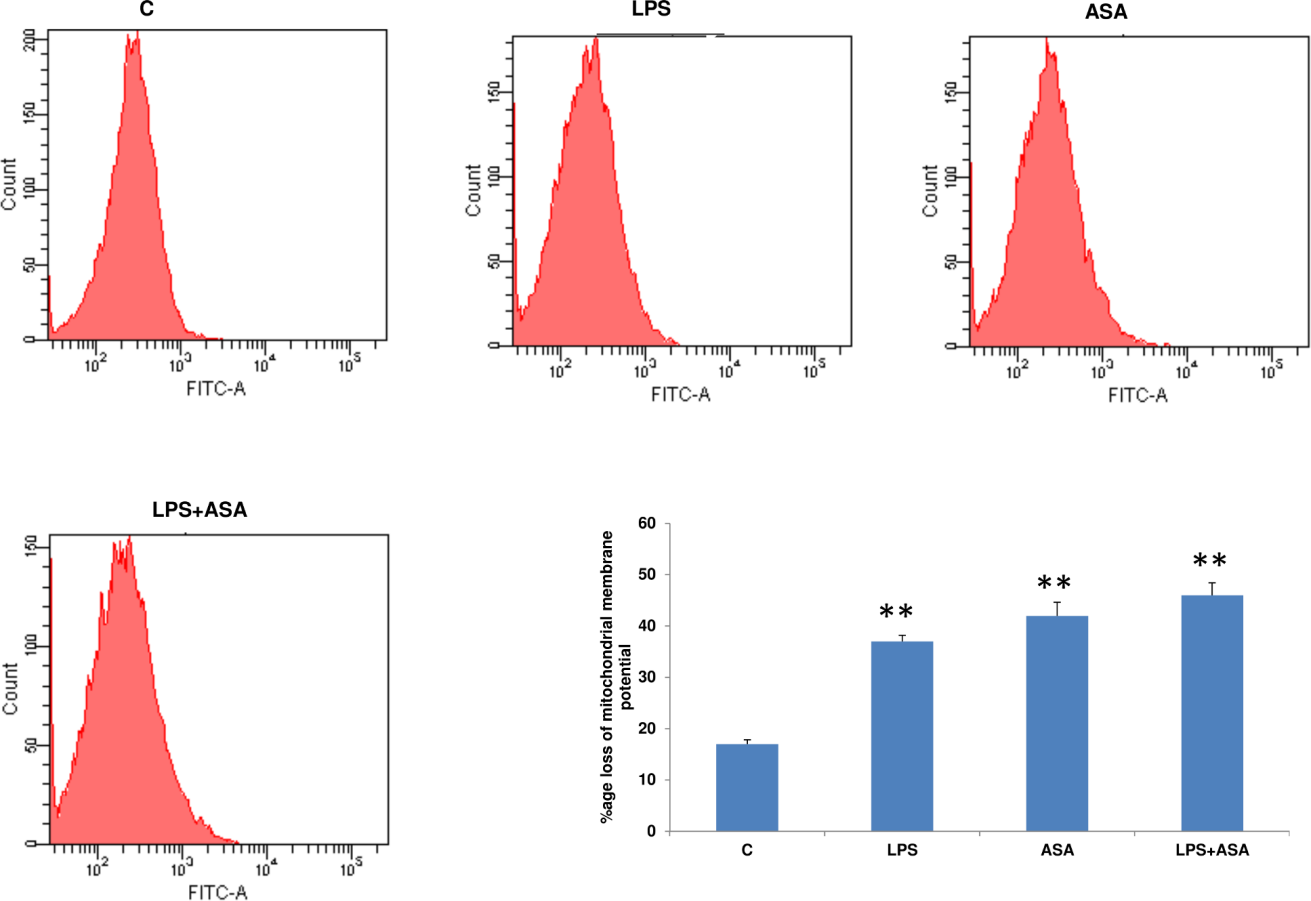
LPS- and ASA-induced alteration in mitochondrial membrane potential. HepG2 cells were cultured to 80% confluence and treated with LPS alone (1μg/ml) for 24h. In some cases, the cells were also treated with 5 mM ASA for 24h alone or in combination with LPS as described above. Mitochondrial membrane potential was measured using a cationic fluorescent dye as described before [13]. A typical histogram representative of at least three experiments showing % loss of mitochondrial membrane potential is shown. Results are expressed as mean +/-SEM of at least three experiments. Asterisks indicate significant difference (**p ≤ 0.001) from control (C).

**Fig 10:**
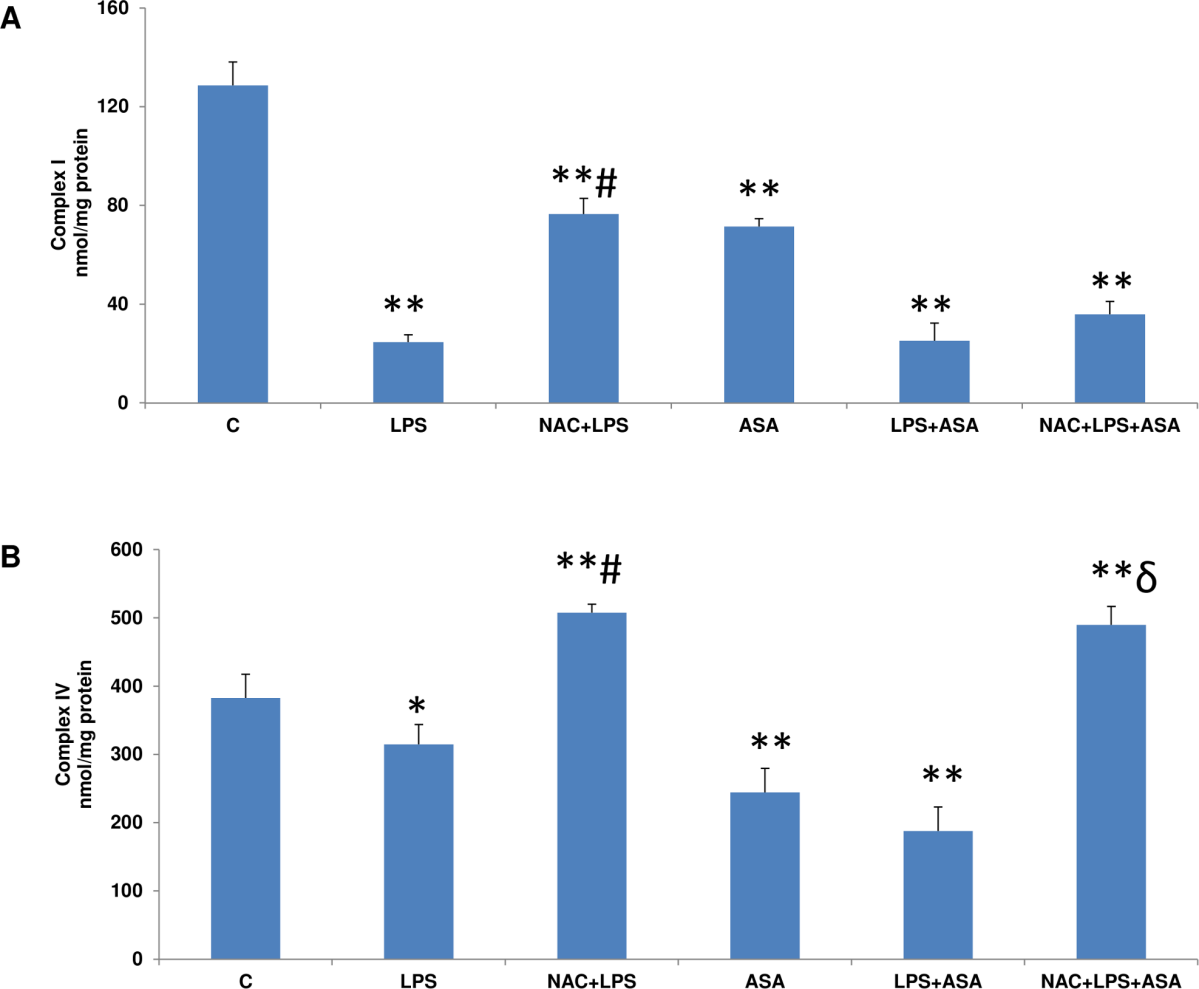
LPS- and ASA-induced alterations in mitochondrial respiratory functions. HepG2 cells were cultured to 80% confluence and treated with LPS alone (1μg/ml) for 24h. In some cases, the cells were also treated with 5 mM ASA for 24h alone or in combination with LPS or in the presence of NAC (10 mM) for 2h prior to LPS treatment, as described above. Mitochondria from control and treated cells were used to assay Complex I (10A) and Complex IV (10B) activities as described before [13]. Results are expressed as mean +/-SEM of at least three experiments. Asterisks indicate significant difference (*p≤0.05, **p ≤0.001) from control (C), ≠p ≤0.05 compared to LPS-treated cells and ɗ p≤0.05 compared to LPS- and ASA-treated cells).

A significant loss in mitochondrial membrane potential (2-3fold) was observed after LPS or ASA treatments alone or in combination (Fig 9). Based on these results, the effects of NAC on mitochondrial enzyme activities were further studied. Complex I activity was significantly inhibited in HepG2cells treated with ASA alone (~45%) and was inhibited even more with LPS alone or in combination with ASA (~80%) (Fig 10A). NAC pre-treatment marginally recovered the activity of this enzyme. Complex IV (cytochrome c oxidase) activity (Fig 10B), on the other hand, was marginally inhibited by LPS alone but was inhibited more significantly with ASA alone or by a combination of LPS and ASA treatments. A significant recovery of enzyme activity was observed in cells pre-treated with NAC.

**Fig 11:**
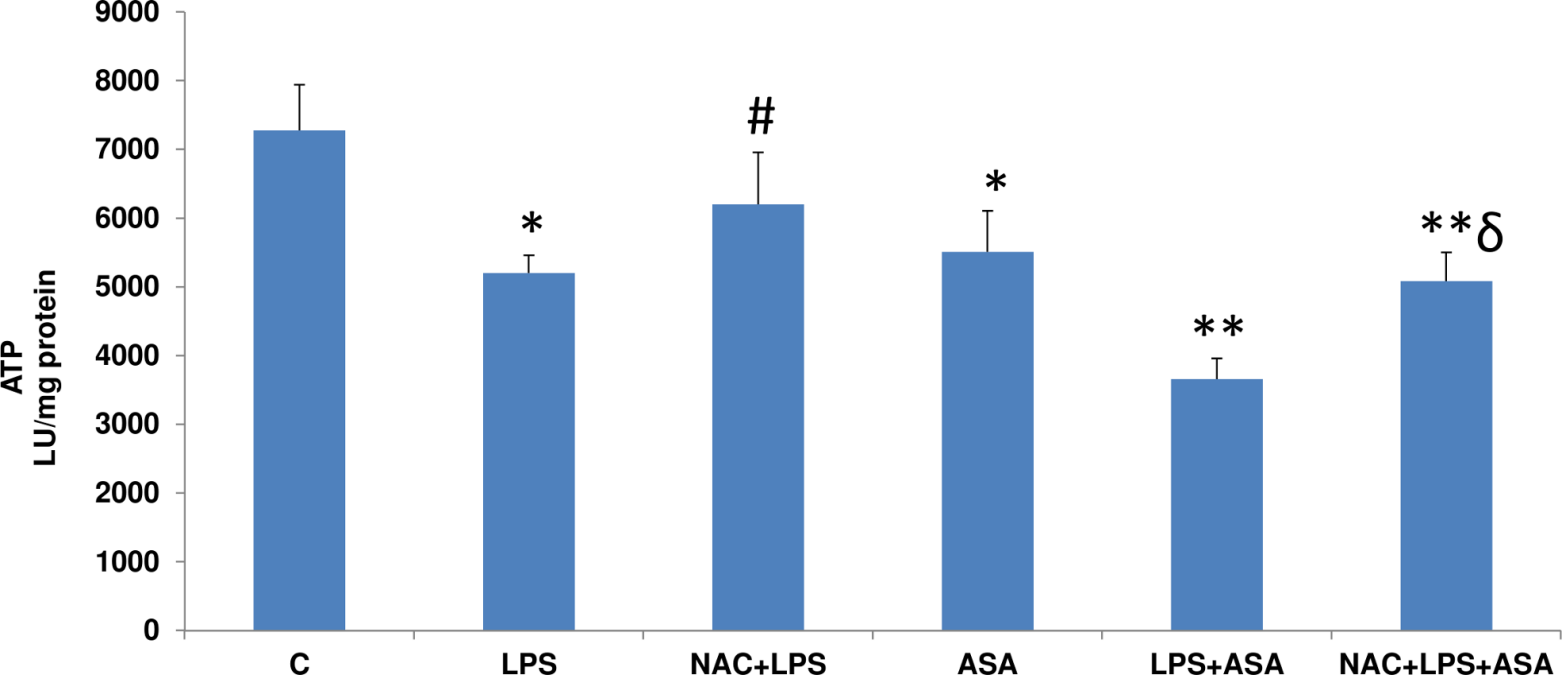
LPS- and ASA-induced alterations on ATP content. HepG2 cells were cultured to 80% confluence and treated with LPS alone (1μg/ml) for 24h. In some cases, the cells were also treated with 5 mM ASA for 24h alone or in combination with LPS or in the presence of NAC (10 mM) for 2h prior to LPS treatment, as described above. Freshly isolated mitochondria from control and treated cells were used to assay the ATP content as described before [13]. Results are expressed as mean +/-SEM of at least three experiments. Asterisks indicate significant difference (*p≤0.05, **p ≤0.001 from control (C), ≠p ≤0.05 compared to LPS-treated cells and ɗ p≤0.05 compared to LPS- and ASA-treated cells).

Treatment of HepG2 cells with LPS or ASA alone or in combination resulted in a significant loss of ATP production (Fig 11) while NAC pre-treatment exhibited a significant recovery in ATP synthesis.

### Effects of LPS and ASA on the expression of redox markers and apoptotic proteins

**Fig 12:**
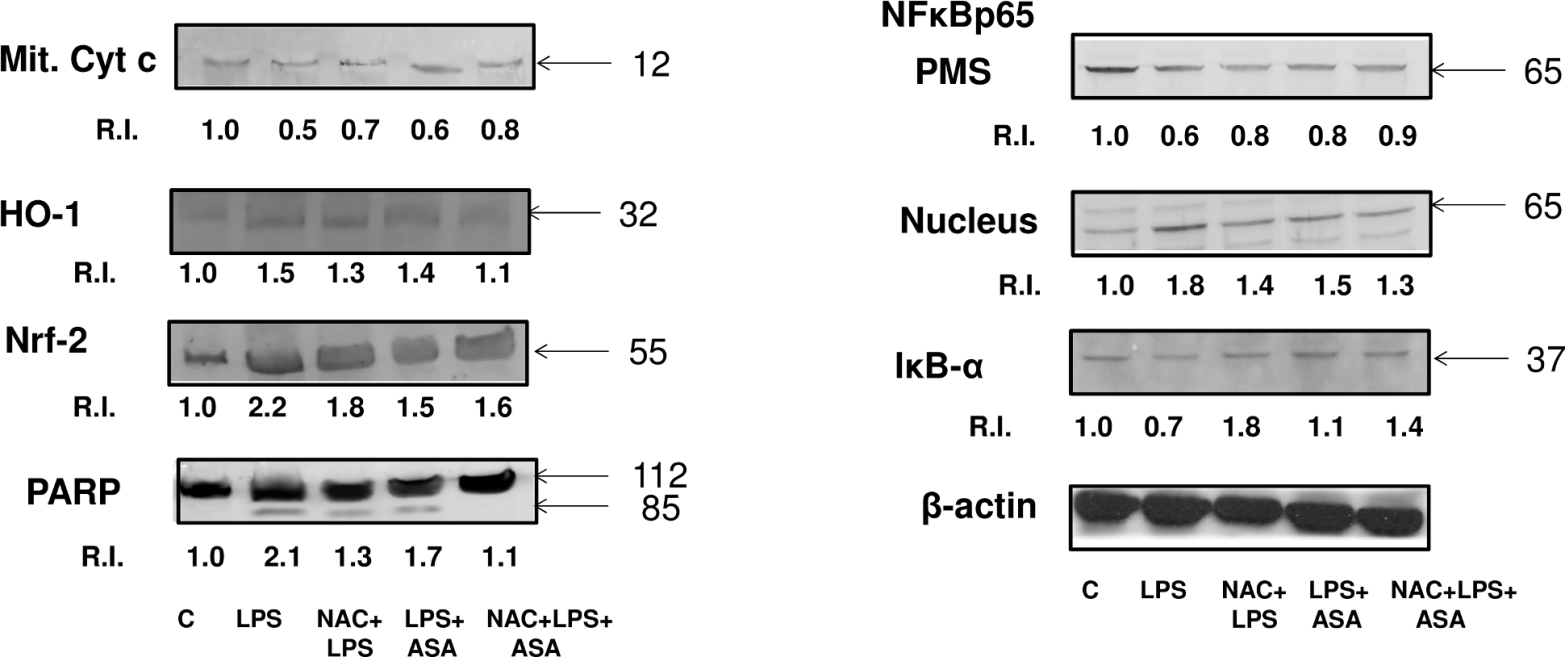
LPS- and ASA-induced alterations in protein expression. HepG2 cells were cultured to 80% confluence and treated with LPS alone (1μg/ml) for 24h or in combination with ASA (5 mM) for 24h with or without NAC (10 mM) for 2h prior to LPS treatment as described above. Proteins (50–100 μg) from cell lysates were separated by SDS-PAGE and transferred on to membranes (Western blot). Transferred proteins were incubated with primary antibodies against cytochrome c, HO-1, Nrf-2, PARP, IκB-α and NF-κBp65 and the specific proteins of interest were visualized as described before [13]. Beta-actin was used as loading control. Results from representative SDS-PAGE/Western blot analysis are shown. The quantitation of protein bands is expressed as relative intensity (R.I) of the protein considering the expression of proteins in control untreated cells as 1.0. Molecular weight markers (kDa) are indicated by arrows.

A 40–50% decrease in the level of mitochondrial cytochrome c content was observed after treatment with LPS alone or in combination with ASA treatment which was partially recovered in the NAC pre-treated cells (Fig 12). A decreased level of mitochondrial cytochrome c content was also observed with different doses of ASA treatment [11]. HO-1 expression was, however, increased after LPS and ASA treatment suggesting an increased oxidative stress, which decreased with NAC treatment. A significant increase in the expression of redox-marker protein, Nrf-2, was also observed when HepG2 cells were treated with LPS alone or in combination with ASA. NAC pre-treatment resulted in a partial recovery of these oxidative stress marker proteins. Increased activation of PARP was also seen after LPS alone or in combination with ASA treatment which was inhibited by NAC treatment suggesting that NAC has some anti-apoptotic effects on HepG2 cells treated with drugs or bacterial toxin. A decrease of cytosolic IκB-α expression and increased nuclear translocation of NF-κBp65 was also observed when HepG2 cells were treated with LPS alone or in combination with ASA. NAC pre-treatment inhibited the translocation. Our previous studies on ASA alone treated HepG2 cells have shown that the increased translocation of cytochrome c resulted in cleavage of caspase 3, accompanied by activation of PARP and a decreased expression of the anti-apoptotic protein, Bcl-2 [11, 12]. These results suggest alterations in redox homeostasis in ASA and LPS-induced HepG2 cells.

## Discussion

The bacterial endotoxin, LPS, regulates the inflammatory and toxic responses in tissues [8, 28]. Alterations in these inflammatory and cytotoxic responses have been reported in different tissues in response to pharmacological interventions. [29–30].We, therefore, have investigated the oxidative stress and associated metabolic complications of LPS and ASA using HepG2 cells in culture. Using HepG2 cells and macrophages, we have provided evidence that both ASA and acetaminophen induce oxidative stress and mitochondrial dysfunction accompanied by apoptosis [11–13, 17–18]. There is considerable evidence that nonsteroidal anti-inflammatory drugs, (NSAIDs), act in synergy with cytokines resulting in chronic inflammatory response and oxidative stress causing hepatotoxicity [3, 29–34].

Aspirin has been reported to activate cytokines production at higher doses while it inhibits their production at a lower dose [33]. We therefore have investigated the effects of ASA and LPS alone or in combination to elucidate the mechanism of cytotoxic responses after endotoxin and drug co-treatment in HepG2 cells. Also, we have investigated the role of NAC, an antioxidant responsible for the maintenance of the GSH pool and a ROS scavenger, in preventing the cytotoxicity caused by LPS alone or in combination with ASA. Our results have indicated an increased cytokine-ROS-dependent oxidative stress and apoptotic response in HepG2 cells towards LPS when treated with ASA which was prevented by NAC treatment. However, ASA appears to have differentially regulated IL-6 and TNF-α in LPS treated HepG2 cells. Earlier reports suggest differential cytokine regulation by aspirin in different cells [32]. Aspirin has been reported to be a potential chemopreventive and antiproliferative NSAID functioning via COX-independent pathways which are presumably associated with increased oxidative stress and mitochondrial dysfunction [35–36]. Our results have also indicated a lower GSH pool, particularly in the mitochondria, and a compromised GSH-dependent redox metabolism after LPS and ASA treatment which was partially or completely recovered after NAC treatment. A synergistic effect of ASA was observed on the increased GSH-conjugating activity by GST in HepG2 cells suggesting enhancement of drug detoxification or exclusion of conjugated drugs/endotoxins from the cells. A significant inhibition of mitochondrial GSH-Px activity and activation of mitochondrial GSH-reductase activities was observed in LPS treated HepG2 cells which were recovered by NAC treatment suggesting the alteration in oxidized GSSG and reduced GSH recycling in this compartment of the cells. ASA treatment alone or in combination with LPS did not exhibit any synergistic effect on the alterations of these enzyme activities.

CYP 2E1 is the most important drug detoxifying enzyme used in the regulation of cellular ROS production [5]. The CYP 2E1 activity was not appreciably altered after LPS treatment alone while it significantly increased when cells were treated with ASA alone or with ASA and LPS in combination. NAC pre-treatment on the other hand, inhibited the enzyme activity in cells treated with LPS alone or in combination with ASA. CYP 3A4 activity, on the other hand, was not affected either by LPS or ASA alone or in combination. NAC pre-treatment inhibited the enzyme activity. These results suggest that the major CYP450 activities are preserved in HepG2 cells and are not affected by LPS/ASA treatments. Lu et al. [5] and Lu and Cederbaum [34] and Cederbaum et al [37] have extensively studied the role of CYP 2E1 in HepG2 cells showing the involvement of enhanced oxidative stress, cytokine-mediated cell signaling and mitochondrial dysfunction in LPS-induced cytotoxicity. Our results also support their observation that LPS or ASA alone or in combination alter ROS-GSH-dependent redox metabolism and apoptosis in HepG2 cells and that CYP2E1 may have some detrimental role in inducing toxicity via oxidative and nitrosative stress. Our previous study using macrophages have also suggested the involvement of CYP 2E1 in ROS production and oxidative stress [13, 17, 18].

Our preliminary results on HepG2 cells have shown that LPS and ASA treatment alone or in combination resulted in increased oxidative and nitrosative stress accompanied by a marked inhibition of ROS-sensitive mitochondrial aconitase [18] suggesting mitochondrial dysfunction. We further confirmed that treatment of macrophages with LPS alone or in combination with ASA induced a loss of mitochondrial membrane potential accompanied by inhibition of respiratory Complex I activity and ATP synthesis [13]. A significant loss of mitochondrial membrane potential was also observed in HepG2 cells after treatment with LPS or ASA alone or in combination followed by reduction in ATP synthesis. The loss of membrane potential in HepG2 cells accompanied by a marked decrease in Complex I and Complex IV activities, which were recovered partially after NAC treatment suggesting some protection of respiratory complexes from oxidative stress. There are reports suggesting that salicylate treatment induces mitochondrial membrane permeability transition causing oxidative stress associated apoptosis [38,39]. Increased ROS production under oxidative stress conditions enhances the glutathionylation of the respiratory complexes and thus alters mitochondrial function and ATP sysnthesis [40]. NAC treatment appears to have protected at least, in part, the oxidative modification of mitochondrial complexes and helped in preserving the mitochondrial function.

Increased expression of redox-sensitive proteins HO-1, Nrf-2 and enhanced nuclear translocation of NF-κB was observed in HepG2 cells after treatment with LPS alone or in combination with ASA. This was accompanied by increased release of mitochondrial cytochrome c and activation of DNA repair enzyme, PARP. NAC treatment appears to have protected the cells from oxidative stress induced apoptosis.

In summary, our results have demonstrated that LPS and ASA treatments have some synergistic effects on HepG2 cells in producing cytokines and oxidative stress mediated mitochondrial dysfunction. NAC treatment protects the cells, at least in part, from LPS/ASA-induced cytotoxicity. These results may have implications in using aspirin therapy in LPS-induced toxicity such as in bacterial infection.
